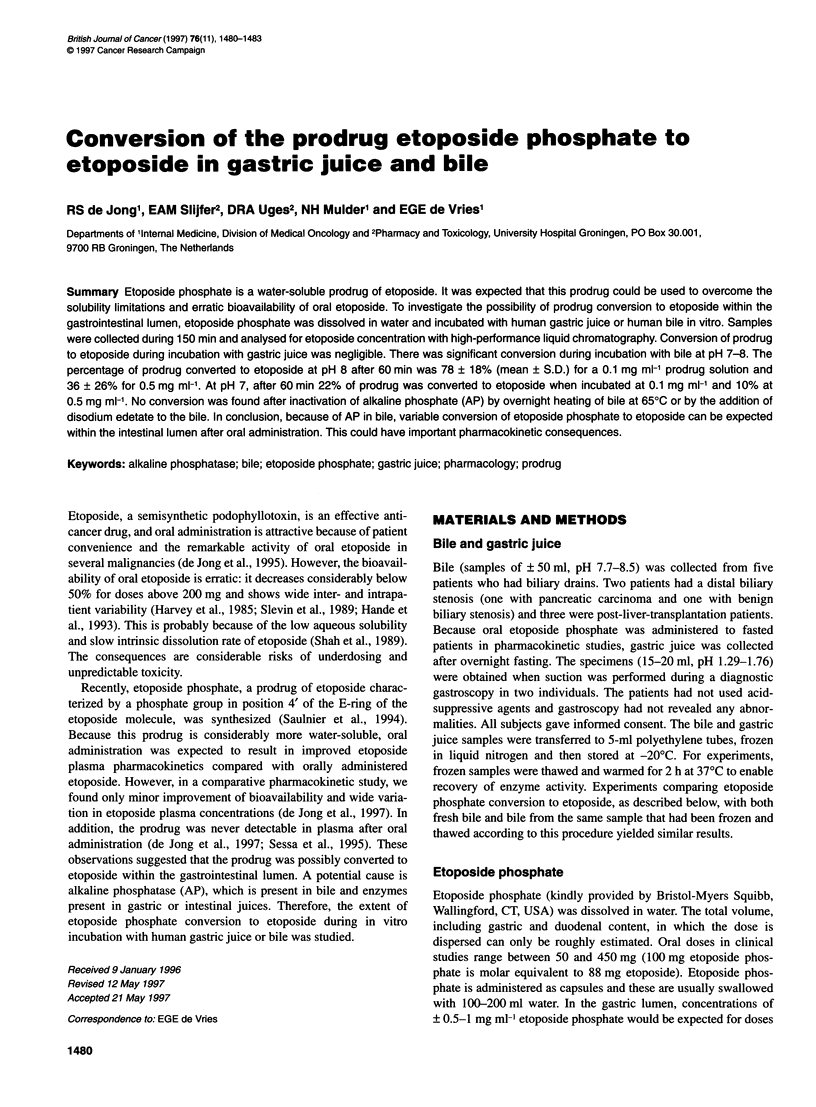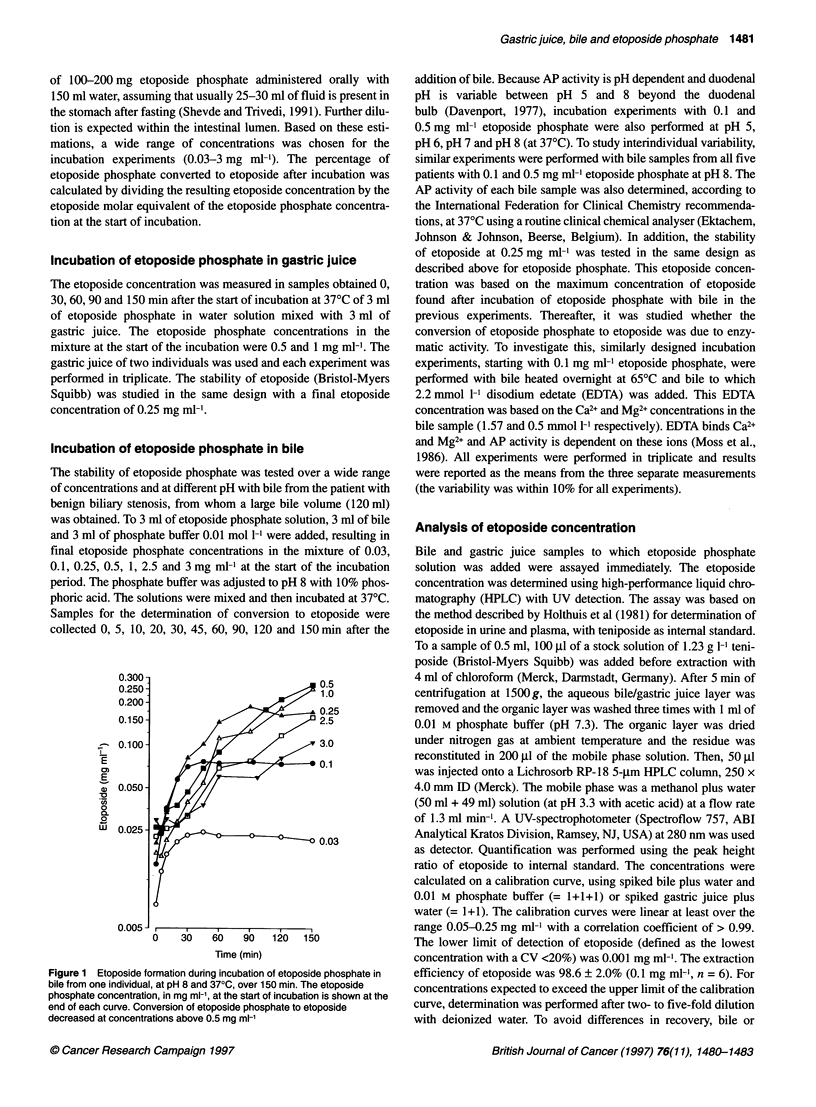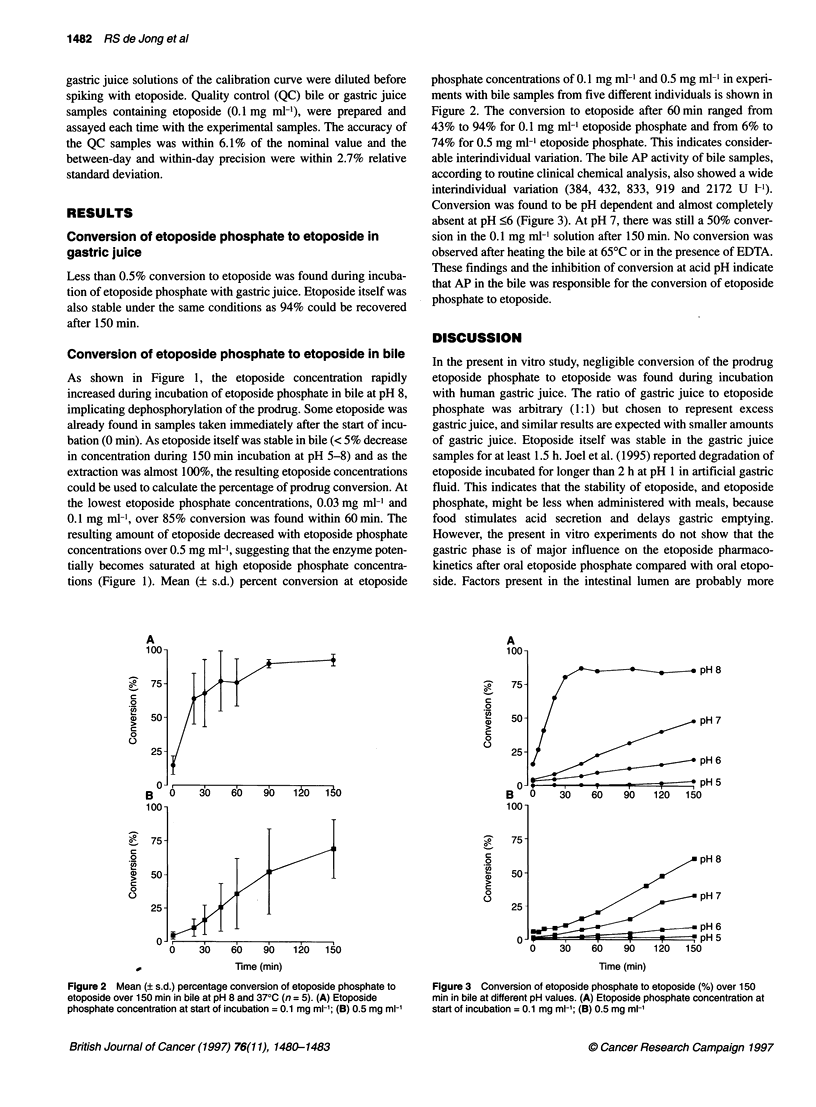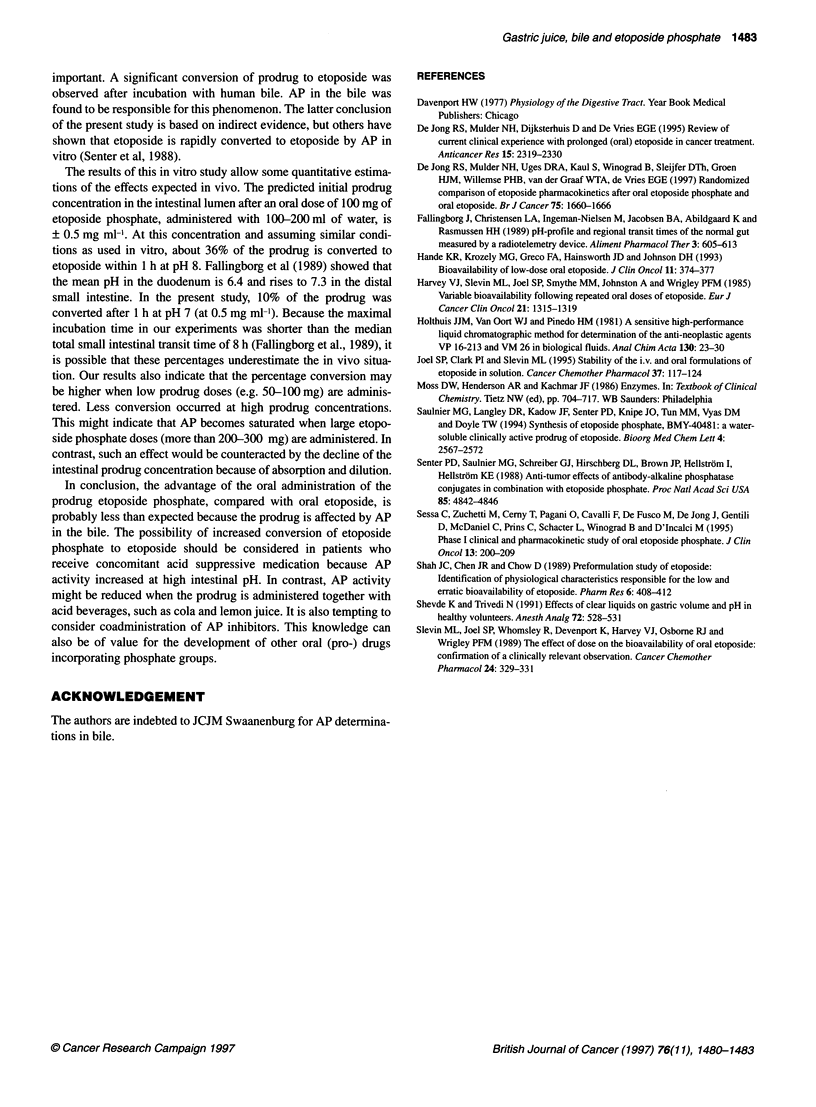# Conversion of the prodrug etoposide phosphate to etoposide in gastric juice and bile.

**DOI:** 10.1038/bjc.1997.581

**Published:** 1997

**Authors:** R. S. de Jong, E. A. Slijfer, D. R. Uges, N. H. Mulder, E. G. de Vries

**Affiliations:** Department of Internal Medicine, University Hospital Groningen, The Netherlands.

## Abstract

Etoposide phosphate is a water-soluble prodrug of etoposide. It was expected that this prodrug could be used to overcome the solubility limitations and erratic bioavailability of oral etoposide. To investigate the possibility of prodrug conversion to etoposide within the gastrointestinal lumen, etoposide phosphate was dissolved in water and incubated with human gastric juice or human bile in vitro. Samples were collected during 150 min and analysed for etoposide concentration with high-performance liquid chromatography. Conversion of prodrug to etoposide during incubation with gastric juice was negligible. There was significant conversion during incubation with bile at pH 7-8. The percentage of prodrug converted to etoposide at pH 8 after 60 min was 78 +/- 18% (mean +/- S.D.) for a 0.1 mg ml-1 prodrug solution and 36 +/- 26% for 0.5 mg ml-1. At pH 7, after 60 min 22% of prodrug was converted to etoposide when incubated at 0.1 mg ml-1 and 10% at 0.5 mg ml-1. No conversion was found after inactivation of alkaline phosphate (AP) by overnight heating of bile at 65 degrees C or by the addition of disodium edetate to the bile. In conclusion, because of AP in bile, variable conversion of etoposide phosphate to etoposide can be expected within the intestinal lumen after oral administration. This could have important pharmacokinetic consequences.


*British Journal of Cancer* (1997) **76**(11), 1480–1483
© 1997 Cancer Research Campaign

### Conversion of the prodrug etoposide phosphate to etoposide in gastric juice and bile

**RS de Jong[1], EAM Slijfer[2], DRA Uges[2], NH Mulder[1] and EGE de Vries[1]**

Departments of [1]Internal Medicine, Division of Medical Oncology and [2]Pharmacy and Toxicology, University Hospital Groningen, PO Box 30.001, 9700 RB Groningen, The Netherlands

**Summary** Etoposide phosphate is a water-soluble prodrug of etoposide. It was expected that this prodrug could be used to overcome the solubility limitations and erratic bioavailability of oral etoposide. To investigate the possibility of prodrug conversion to etoposide within the gastrointestinal lumen, etoposide phosphate was dissolved in water and incubated with human gastric juice or human bile in vitro. Samples were collected during 150 min and analysed for etoposide concentration with high-performance liquid chromatography. Conversion of prodrug to etoposide during incubation with gastric juice was negligible. There was significant conversion during incubation with bile at pH 7–8. The percentage of prodrug converted to etoposide at pH 8 after 60 min was 78 ± 18% (mean ± S.D.) for a 0.1 mg $ml^{-1}$ prodrug solution and 36 ± 26% for 0.5 mg $ml^{-1}$. At pH 7, after 60 min 22% of prodrug was converted to etoposide when incubated at 0.1 mg $ml^{-1}$ and 10% at 0.5 mg $ml^{-1}$. No conversion was found after inactivation of alkaline phosphate (AP) by overnight heating of bile at 65°C or by the addition of disodium edetate to the bile. In conclusion, because of AP in bile, variable conversion of etoposide phosphate to etoposide can be expected within the intestinal lumen after oral administration. This could have important pharmacokinetic consequences.

**Keywords:** alkaline phosphatase; bile; etoposide phosphate; gastric juice; pharmacology; prodrug

Etoposide, a semisynthetic podophyllotoxin, is an effective anticancer drug, and oral administration is attractive because of patient convenience and the remarkable activity of oral etoposide in several malignancies (de Jong et al., 1995). However, the bioavailability of oral etoposide is erratic: it decreases considerably below 50% for doses above 200 mg and shows wide inter- and intrapatient variability (Harvey et al., 1985; Slevin et al., 1989; Hande et al., 1993). This is probably because of the low aqueous solubility and slow intrinsic dissolution rate of etoposide (Shah et al., 1989). The consequences are considerable risks of underdosing and unpredictable toxicity.

Recently, etoposide phosphate, a prodrug of etoposide characterized by a phosphate group in position 4′ of the E-ring of the etoposide molecule, was synthesized (Saulnier et al., 1994). Because this prodrug is considerably more water-soluble, oral administration was expected to result in improved etoposide plasma pharmacokinetics compared with orally administered etoposide. However, in a comparative pharmacokinetic study, we found only minor improvement of bioavailability and wide variation in etoposide plasma concentrations (de Jong et al., 1997). In addition, the prodrug was never detectable in plasma after oral administration (de Jong et al., 1997; Sessa et al., 1995). These observations suggested that the prodrug was possibly converted to etoposide within the gastrointestinal lumen. A potential cause is alkaline phosphatase (AP), which is present in bile and enzymes present in gastric or intestinal juices. Therefore, the extent of etoposide phosphate conversion to etoposide during in vitro incubation with human gastric juice or bile was studied.

*Received 9 January 1996*
*Revised 12 May 1997*
*Accepted 21 May 1997*

*Correspondence to:* EGE de Vries

### MATERIALS AND METHODS

### Bile and gastric juice

Bile (samples of ± 50 ml, pH 7.7–8.5) was collected from five patients who had biliary drains. Two patients had a distal biliary stenosis (one with pancreatic carcinoma and one with benign biliary stenosis) and three were post-liver-transplantation patients. Because oral etoposide phosphate was administered to fasted patients in pharmacokinetic studies, gastric juice was collected after overnight fasting. The specimens (15–20 ml, pH 1.29–1.76) were obtained when suction was performed during a diagnostic gastroscopy in two individuals. The patients had not used acid-suppressive agents and gastroscopy had not revealed any abnormalities. All subjects gave informed consent. The bile and gastric juice samples were transferred to 5-ml polyethylene tubes, frozen in liquid nitrogen and then stored at –20°C. For experiments, frozen samples were thawed and warmed for 2 h at 37°C to enable recovery of enzyme activity. Experiments comparing etoposide phosphate conversion to etoposide, as described below, with both fresh bile and bile from the same sample that had been frozen and thawed according to this procedure yielded similar results.

### Etoposide phosphate

Etoposide phosphate (kindly provided by Bristol-Myers Squibb, Wallingford, CT, USA) was dissolved in water. The total volume, including gastric and duodenal content, in which the dose is dispersed can only be roughly estimated. Oral doses in clinical studies range between 50 and 450 mg (100 mg etoposide phosphate is molar equivalent to 88 mg etoposide). Etoposide phosphate is administered as capsules and these are usually swallowed with 100–200 ml water. In the gastric lumen, concentrations of ± 0.5–1 mg $ml^{-1}$ etoposide phosphate would be expected for doses of 100–200 mg etoposide phosphate administered orally with 150 ml water, assuming that usually 25–30 ml of fluid is present in the stomach after fasting (Shevde and Trivedi, 1991). Further dilution is expected within the intestinal lumen. Based on these estimations, a wide range of concentrations was chosen for the incubation experiments (0.03–3 mg $ml^{-1}$). The percentage of etoposide phosphate converted to etoposide after incubation was calculated by dividing the resulting etoposide concentration by the etoposide molar equivalent of the etoposide phosphate concentration at the start of incubation.

### Incubation of etoposide phosphate in gastric juice

The etoposide concentration was measured in samples obtained 0, 30, 60, 90 and 150 min after the start of incubation at 37°C of 3 ml of etoposide phosphate in water solution mixed with 3 ml of gastric juice. The etoposide phosphate concentrations in the mixture at the start of the incubation were 0.5 and 1 mg $ml^{-1}$. The gastric juice of two individuals was used and each experiment was performed in triplicate. The stability of etoposide (Bristol-Myers Squibb) was studied in the same design with a final etoposide concentration of 0.25 mg $ml^{-1}$.

### Incubation of etoposide phosphate in bile

The stability of etoposide phosphate was tested over a wide range of concentrations and at different pH with bile from the patient with benign biliary stenosis, from whom a large bile volume (120 ml) was obtained. To 3 ml of etoposide phosphate solution, 3 ml of bile and 3 ml of phosphate buffer 0.01 mol $l^{-1}$ were added, resulting in final etoposide phosphate concentrations in the mixture of 0.03, 0.1, 0.25, 0.5, 1, 2.5 and 3 mg $ml^{-1}$ at the start of the incubation period. The phosphate buffer was adjusted to pH 8 with 10% phosphoric acid. The solutions were mixed and then incubated at 37°C. Samples for the determination of conversion to etoposide were collected 0, 5, 10, 20, 30, 45, 60, 90, 120 and 150 min after the

**Figure 1** Etoposide formation during incubation of etoposide phosphate in bile from one individual, at pH 8 and 37°C, over 150 min. The etoposide phosphate concentration, in mg $ml^{-1}$, at the start of incubation is shown at the end of each curve. Conversion of etoposide phosphate to etoposide decreased at concentrations above 0.5 mg $ml^{-1}$

addition of bile. Because AP activity is pH dependent and duodenal pH is variable between pH 5 and 8 beyond the duodenal bulb (Davenport, 1977), incubation experiments with 0.1 and 0.5 mg $ml^{-1}$ etoposide phosphate were also performed at pH 5, pH 6, pH 7 and pH 8 (at 37°C). To study interindividual variability, similar experiments were performed with bile samples from all five patients with 0.1 and 0.5 mg $ml^{-1}$ etoposide phosphate at pH 8. The AP activity of each bile sample was also determined, according to the International Federation for Clinical Chemistry recommendations, at 37°C using a routine clinical chemical analyser (Ektachem, Johnson & Johnson, Beerse, Belgium). In addition, the stability of etoposide at 0.25 mg $ml^{-1}$ was tested in the same design as described above for etoposide phosphate. This etoposide concentration was based on the maximum concentration of etoposide found after incubation of etoposide phosphate with bile in the previous experiments. Thereafter, it was studied whether the conversion of etoposide phosphate to etoposide was due to enzymatic activity. To investigate this, similarly designed incubation experiments, starting with 0.1 mg $ml^{-1}$ etoposide phosphate, were performed with bile heated overnight at 65°C and bile to which 2.2 mmol $l^{-1}$ disodium edetate (EDTA) was added. This EDTA concentration was based on the $Ca^{2+}$ and $Mg^{2+}$ concentrations in the bile sample (1.57 and 0.5 mmol $l^{-1}$ respectively). EDTA binds $Ca^{2+}$ and $Mg^{2+}$ and AP activity is dependent on these ions (Moss et al., 1986). All experiments were performed in triplicate and results were reported as the means from the three separate measurements (the variability was within 10% for all experiments).

### Analysis of etoposide concentration

Bile and gastric juice samples to which etoposide phosphate solution was added were assayed immediately. The etoposide concentration was determined using high-performance liquid chromatography (HPLC) with UV detection. The assay was based on the method described by Holthuis et al (1981) for determination of etoposide in urine and plasma, with teniposide as internal standard. To a sample of 0.5 ml, 100 µl of a stock solution of 1.23 g $l^{-1}$ teniposide (Bristol-Myers Squibb) was added before extraction with 4 ml of chloroform (Merck, Darmstadt, Germany). After 5 min of centrifugation at 1500 *g*, the aqueous bile/gastric juice layer was removed and the organic layer was washed three times with 1 ml of 0.01 M phosphate buffer (pH 7.3). The organic layer was dried under nitrogen gas at ambient temperature and the residue was reconstituted in 200 µl of the mobile phase solution. Then, 50 µl was injected onto a Lichrosorb RP-18 5-µm HPLC column, 250 × 4.0 mm ID (Merck). The mobile phase was a methanol plus water (50 ml + 49 ml) solution (at pH 3.3 with acetic acid) at a flow rate of 1.3 ml $min^{-1}$. A UV-spectrophotometer (Spectroflow 757, ABI Analytical Kratos Division, Ramsey, NJ, USA) at 280 nm was used as detector. Quantification was performed using the peak height ratio of etoposide to internal standard. The concentrations were calculated on a calibration curve, using spiked bile plus water and 0.01 M phosphate buffer (= 1+1+1) or spiked gastric juice plus water (= 1+1). The calibration curves were linear at least over the range 0.05–0.25 mg $ml^{-1}$ with a correlation coefficient of > 0.99. The lower limit of detection of etoposide (defined as the lowest concentration with a CV <20%) was 0.001 mg $ml^{-1}$. The extraction efficiency of etoposide was 98.6 ± 2.0% (0.1 mg $ml^{-1}$, $n$ = 6). For concentrations expected to exceed the upper limit of the calibration curve, determination was performed after two- to five-fold dilution with deionized water. To avoid differences in recovery, bile or

© Cancer Research Campaign 1997

gastric juice solutions of the calibration curve were diluted before spiking with etoposide. Quality control (QC) bile or gastric juice samples containing etoposide (0.1 mg $ml^{-1}$), were prepared and assayed each time with the experimental samples. The accuracy of the QC samples was within 6.1% of the nominal value and the between-day and within-day precision were within 2.7% relative standard deviation.

### RESULTS

### Conversion of etoposide phosphate to etoposide in gastric juice

Less than 0.5% conversion to etoposide was found during incubation of etoposide phosphate with gastric juice. Etoposide itself was also stable under the same conditions as 94% could be recovered after 150 min.

### Conversion of etoposide phosphate to etoposide in bile

As shown in Figure 1, the etoposide concentration rapidly increased during incubation of etoposide phosphate in bile at pH 8, implicating dephosphorylation of the prodrug. Some etoposide was already found in samples taken immediately after the start of incubation (0 min). As etoposide itself was stable in bile (< 5% decrease in concentration during 150 min incubation at pH 5–8) and as the extraction was almost 100%, the resulting etoposide concentrations could be used to calculate the percentage of prodrug conversion. At the lowest etoposide phosphate concentrations, 0.03 mg $ml^{-1}$ and 0.1 mg $ml^{-1}$, over 85% conversion was found within 60 min. The resulting amount of etoposide decreased with etoposide phosphate concentrations over 0.5 mg $ml^{-1}$, suggesting that the enzyme potentially becomes saturated at high etoposide phosphate concentrations (Figure 1). Mean (± s.d.) percent conversion at etoposide phosphate concentrations of 0.1 mg $ml^{-1}$ and 0.5 mg $ml^{-1}$ in experiments with bile samples from five different individuals is shown in Figure 2. The conversion to etoposide after 60 min ranged from 43% to 94% for 0.1 mg $ml^{-1}$ etoposide phosphate and from 6% to 74% for 0.5 mg $ml^{-1}$ etoposide phosphate. This indicates considerable interindividual variation. The bile AP activity of bile samples, according to routine clinical chemical analysis, also showed a wide interindividual variation (384, 432, 833, 919 and 2172 U $l^{-1}$). Conversion was found to be pH dependent and almost completely absent at pH ≤6 (Figure 3). At pH 7, there was still a 50% conversion in the 0.1 mg $ml^{-1}$ solution after 150 min. No conversion was observed after heating the bile at 65°C or in the presence of EDTA. These findings and the inhibition of conversion at acid pH indicate that AP in the bile was responsible for the conversion of etoposide phosphate to etoposide.

### DISCUSSION

In the present in vitro study, negligible conversion of the prodrug etoposide phosphate to etoposide was found during incubation with human gastric juice. The ratio of gastric juice to etoposide phosphate was arbitrary (1:1) but chosen to represent excess gastric juice, and similar results are expected with smaller amounts of gastric juice. Etoposide itself was stable in the gastric juice samples for at least 1.5 h. Joel et al. (1995) reported degradation of etoposide incubated for longer than 2 h at pH 1 in artificial gastric fluid. This indicates that the stability of etoposide, and etoposide phosphate, might be less when administered with meals, because food stimulates acid secretion and delays gastric emptying. However, the present in vitro experiments do not show that the gastric phase is of major influence on the etoposide pharmacokinetics after oral etoposide phosphate compared with oral etoposide. Factors present in the intestinal lumen are probably more

**Figure 2** Mean (± s.d.) percentage conversion of etoposide phosphate to etoposide over 150 min in bile at pH 8 and 37°C (*n* = 5). (**A**) Etoposide phosphate concentration at start of incubation = 0.1 mg $ml^{-1}$; (**B**) 0.5 mg $ml^{-1}$

**Figure 3** Conversion of etoposide phosphate to etoposide (%) over 150 min in bile at different pH values. (**A**) Etoposide phosphate concentration at start of incubation = 0.1 mg $ml^{-1}$; (**B**) 0.5 mg $ml^{-1}$

© Cancer Research Campaign 1997

important. A significant conversion of prodrug to etoposide was observed after incubation with human bile. AP in the bile was found to be responsible for this phenomenon. The latter conclusion of the present study is based on indirect evidence, but others have shown that etoposide is rapidly converted to etoposide by AP in vitro (Senter et al, 1988).

The results of this in vitro study allow some quantitative estimations of the effects expected in vivo. The predicted initial prodrug concentration in the intestinal lumen after an oral dose of 100 mg of etoposide phosphate, administered with 100–200 ml of water, is ± 0.5 mg $ml^{-1}$. At this concentration and assuming similar conditions as used in vitro, about 36% of the prodrug is converted to etoposide within 1 h at pH 8. Fallingborg et al (1989) showed that the mean pH in the duodenum is 6.4 and rises to 7.3 in the distal small intestine. In the present study, 10% of the prodrug was converted after 1 h at pH 7 (at 0.5 mg $ml^{-1}$). Because the maximal incubation time in our experiments was shorter than the median total small intestinal transit time of 8 h (Fallingborg et al., 1989), it is possible that these percentages underestimate the in vivo situation. Our results also indicate that the percentage conversion may be higher when low prodrug doses (e.g. 50–100 mg) are administered. Less conversion occurred at high prodrug concentrations. This might indicate that AP becomes saturated when large etoposide phosphate doses (more than 200–300 mg) are administered. In contrast, such an effect would be counteracted by the decline of the intestinal prodrug concentration because of absorption and dilution.

In conclusion, the advantage of the oral administration of the prodrug etoposide phosphate, compared with oral etoposide, is probably less than expected because the prodrug is affected by AP in the bile. The possibility of increased conversion of etoposide phosphate to etoposide should be considered in patients who receive concomitant acid suppressive medication because AP activity increased at high intestinal pH. In contrast, AP activity might be reduced when the prodrug is administered together with acid beverages, such as cola and lemon juice. It is also tempting to consider coadministration of AP inhibitors. This knowledge can also be of value for the development of other oral (pro-) drugs incorporating phosphate groups.

### ACKNOWLEDGEMENT

The authors are indebted to JCJM Swaanenburg for AP determinations in bile.

### REFERENCES

Davenport HW (1977) *Physiology of the Digestive Tract.* Year Book Medical Publishers: Chicago

De Jong RS, Mulder NH, Dijksterhuis D and De Vries EGE (1995) Review of current clinical experience with prolonged (oral) etoposide in cancer treatment. *Anticancer Res* **15**: 2319–2330

De Jong RS, Mulder NH, Uges DRA, Kaul S, Winograd B, Sleijfer DTh, Groen HJM, Willemse PHB, van der Graaf WTA, de Vries EGE (1997) Randomized comparison of etoposide pharmacokinetics after oral etoposide phosphate and oral etoposide. *Br J Cancer* **75**: 1660–1666

Fallingborg J, Christensen LA, Ingeman-Nielsen M, Jacobsen BA, Abildgaard K and Rasmussen HH (1989) pH-profile and regional transit times of the normal gut measured by a radiotelemetry device. *Aliment Pharmacol Ther* **3**: 605–613

Hande KR, Krozely MG, Greco FA, Hainsworth JD and Johnson DH (1993) Bioavailability of low-dose oral etoposide. *J Clin Oncol* **11**: 374–377

Harvey VJ, Slevin ML, Joel SP, Smythe MM, Johnston A and Wrigley PFM (1985) Variable bioavailability following repeated oral doses of etoposide. *Eur J Cancer Clin Oncol* **21**: 1315–1319

Holthuis JJM, Van Oort WJ and Pinedo HM (1981) A sensitive high-performance liquid chromatographic method for determination of the anti-neoplastic agents VP 16-213 and VM 26 in biological fluids. *Anal Chim Acta* **130**: 23–30

Joel SP, Clark PI and Slevin ML (1995) Stability of the i.v. and oral formulations of etoposide in solution. *Cancer Chemother Pharmacol* **37**: 117–124

Moss DW, Henderson AR and Kachmar JF (1986) Enzymes. In: *Textbook of Clinical Chemistry.* Tietz NW (ed), pp. 704–717. WB Saunders: Philadelphia

Saulnier MG, Langley DR, Kadow JF, Senter PD, Knipe JO, Tun MM, Vyas DM and Doyle TW (1994) Synthesis of etoposide phosphate, BMY-40481: a water-soluble clinically active prodrug of etoposide. *Bioorg Med Chem Lett* **4**: 2567–2572

Senter PD, Saulnier MG, Schreiber GJ, Hirschberg DL, Brown JP, Hellström I, Hellström KE (1988) Anti-tumor effects of antibody-alkaline phosphatase conjugates in combination with etoposide phosphate. *Proc Natl Acad Sci USA* **85**: 4842–4846

Sessa C, Zuchetti M, Cerny T, Pagani O, Cavalli F, De Fusco M, De Jong J, Gentili D, McDaniel C, Prins C, Schacter L, Winograd B and D'Incalci M (1995) Phase I clinical and pharmacokinetic study of oral etoposide phosphate. *J Clin Oncol* **13**: 200–209

Shah JC, Chen JR and Chow D (1989) Preformulation study of etoposide: Identification of physiological characteristics responsible for the low and erratic bioavailability of etoposide. *Pharm Res* **6**: 408–412

Shevde K and Trivedi N (1991) Effects of clear liquids on gastric volume and pH in healthy volunteers. *Anesth Analg* **72**: 528–531

Slevin ML, Joel SP, Whomsley R, Devenport K, Harvey VJ, Osborne RJ and Wrigley PFM (1989) The effect of dose on the bioavailability of oral etoposide: confirmation of a clinically relevant observation. *Cancer Chemother Pharmacol* **24**: 329–331

© Cancer Research Campaign 1997